# In silico identification of enhancers on the basis of a combination of transcription factor binding motif occurrences

**DOI:** 10.1038/srep32476

**Published:** 2016-09-01

**Authors:** Yaping Fang, Yunlong Wang, Qin Zhu, Jia Wang, Guoliang Li

**Affiliations:** 1 Agricultural Bioinformatics Key Laboratory of Hubei Province, Huazhong Agricultural University, Wuhan 430070, China; 2 College of Informatics, Huazhong Agricultural University, Wuhan 430070, China; 3 National Key Laboratory of Crop Genetic Improvement, Huazhong Agricultural University, Wuhan 430070, China

## Abstract

Enhancers interact with gene promoters and form chromatin looping structures that serve important functions in various biological processes, such as the regulation of gene transcription and cell differentiation. However, enhancers are difficult to identify because they generally do not have fixed positions or consensus sequence features, and biological experiments for enhancer identification are costly in terms of labor and expense. In this work, several models were built by using various sequence-based feature sets and their combinations for enhancer prediction. The selected features derived from a recursive feature elimination method showed that the model using a combination of 141 transcription factor binding motif occurrences from 1,422 transcription factor position weight matrices achieved a favorably high prediction accuracy superior to that of other reported methods. The models demonstrated good prediction accuracy for different enhancer datasets obtained from different cell lines/tissues. In addition, prediction accuracy was further improved by integration of chromatin state features. Our method is complementary to wet-lab experimental methods and provides an additional method to identify enhancers.

Understanding eukaryotic gene transcription and regulation is an important task in the post-genomic era. Gene transcription and regulation is a complex and multi-stage process involving many factors, such as enhancers and gene promoters. Enhancers are a class of non-coding regulatory DNA elements that interact with distal and proximal gene promoters with the help of activators or mediators. Since the first enhancer was discovered in SV40 DNA in 1981, many enhancers from different species have been identified^1^. It is now widely accepted that enhancers are present extensively in higher eukaryotes^1^,^2^. Enhancers play important roles in biological processes, such as gene transcription and regulation^3^, determination of the three-dimensional structure of chromatin^4^,^5^,^6^, cell differentiation^6^ and diseases^7^,^8^. Recent studies have shown that enhancers are complex regulatory elements that are associated with epigenetic information, such as histone methylation, open chromatin regions and transcription factor (TF) binding sites^9^,^10^. For example, enhancers usually overlap with open chromatin regions and are associated with certain chromatin state^11^,^12^. Enhancers are generally classified into two groups according to their activities. The first group comprises the active enhancers, which are usually characterized by histone Lys4 mono-methylation (H3K4me1) and histone Lys27 acetylation (H3K27ac). The other group comprises the poised enhancers, which are characterized by H3K4me1 and H3K27me3^13^. In addition, enhancers may be transcribed into RNA transcripts^14^ designated “eRNAs”. These eRNAs promote the formation of loops between enhancers and promoters during gene regulation.

Traditionally, enhancers have been identified through enhancer trap techniques^10^ using reporter genes in model organisms, such as humans, mice, and *C. elegans*^15^. These experiments are often high-cost, time-consuming, labor-intensive and low-throughput. Owing to the large advantages of current sequencing technology, the functions of enhancers can now be identified and investigated via whole genome sequencing. Generally, two high-throughput experiment methods can be used to identify enhancers in whole genome studies. The first method is to identify enhancers by investigating open chromatin regions via DNase I hypersensitivity mapping^16^,^17^. However, open chromatin regions contain insulators and promoters in addition to enhancers. The second method is to identify enhancers from the DNA binding sites of proteins via chromatin immunoprecipitation coupled with massively parallel sequencing (ChIP-Seq) technology^18^. The immunoprecipitated proteins may be various TFs, such as p300 (also called EP300 or E1A binding protein p300), as well as CBP proteins (also known as CREB-binding protein or CREBBP) and histones. However, owing to the costs and resources required for ChIP-Seq experiments, this methodology can identify only a fraction of enhancers. Thus, there is a need to develop high-throughput and rapid in silico methods to reliably detect enhancers in the entire genomes.

In addition to the above wet-lab-based methods, several computation-based methods have been developed to predict enhancers. These methods for enhancer prediction generally fall into the following two categories: those using chromatin state measurements as features^16^ and those using DNA sequence features^19^,^20^,^21^. For example, Rajagopal *et al*.^16^ have developed a random forest model to predict enhancers on the basis of 24 histone modifications. Zhu *et al*.^13^ have constructed a model to predict enhancers on the basis of histone modifications with a logistic regression algorithm. Podsiadlo *et al*.^22^ have built a model to predict active enhancers on the basis of histone modifications and collective motif data. Taher *et al*.^23^ have developed a model to predict distal enhancers by using the sequence signatures of promoters and the Support Vector Machine (SVM) algorithm. Ghandi *et al*.^21^ have developed a model to predict enhancers on the basis of gapped k-mer features. Erwin *et al*.^24^ have developed an SVM model to predict enhancers by integrating various data, such as evolutionary conservation, regulatory protein binding, chromatin modifications, and DNA sequence motifs. Recently, Whitaker *et al*.^25^ have reviewed current progress in terms of the prediction and annotation of enhancers. However, there are several limitations to these methods. First, not all annotation data, such as histone modifications, are readily available for a particular cell type or tissue, thus restricting the use of these methods. Second, it is unclear which feature groups used in the above methods are important to the performance of the models.

To answer these questions, we built models based on various types of sequence information to identify enhancers. The feature groups included DNA properties, k-mers, chromatin state and 1,422 transcription factor binding motif occurrences. A recursive feature elimination method was used to select the informative features for each feature group and their combinations. The results showed that with sequence-based features, our method using the combination of TF binding motif occurrences was superior to other reported methods, with the performance values as follows: sensitivity (Se) 0.8473, specificity (Sp) 0.9753, accuracy (ACC) 0.9113, area under the receiver operating characteristic (ROC) curve (AUC) 0.9698 and Mathews correlation coefficient (MCC) 0.8293. In addition to the sequences around enhancers, we also included histone modification ChIP-Seq datasets when they were available. The model incorporating TF binding motif occurrence and chromatin state achieved the following performance: Se 0.955, Sp 0.95, ACC 0.9525, AUC 0.989 and MCC 0.9051. The results indicate that enhancers can be predicted by using only the sequence-based TF binding motif occurrence model, which can be further improved by the addition of chromatin state features. It is reveals that complementary effects are present not only in the TFs and chromatin states but also between them. Compared with previous methods, our method demonstrates superior performance and should be a useful methodology for the study of enhancers.

## Results

### Performance of individual sequence-based feature groups

A previous publication has indicated that GC content is important for splicing and transcription regulation^26^. To verify the significance of GC content present in both enhancers and random genomic sequences, GC distributions were calculated and compared. The results showed that there were no statistically significant difference between these two groups (*p*-value = 0.3696 with paired Student *t*-test).

Because different feature groups generally represent different types of information regarding enhancers and control regions, it is necessary to systematically evaluate the performance of different feature groups and their combinations. The sequence-based feature groups and their combinations used to build models, as well as specific results, are given in Table 1. For single feature groups, the performance in decreasing order was: TF binding motif occurrence, DNA properties and k-mers. The model based on TF binding motif occurrence demonstrated the best performance with an ACC of 0.8993, MCC of 0.8087 and AUC of 0.9687. These results indicate that TF binding motifs play a vital role in the identification of enhancers and suggest that TFs are important for enhancer function. The model based on DNA properties also demonstrated acceptable performance. This interesting result indicates that DNA structural information also plays a role in the identification of enhancers. Although previous publications have indicated that k-mer information is important for the identification of genes and regulatory sequences^13^,^27^, the results in this work suggest that k-mer information has little effect on the identification of enhancers. The reasons for this may be that only a limited set of k-mers were considered, and those k-mers were not specifically developed for the prediction of enhancers.

Combinations of different sequence-based feature groups were tested for enhancer identification. The results show that there is no improvement in prediction performance (Table 1), when compared with the results from TF binding motif occurrence model alone. These findings suggest that TF binding motif occurrence exerts a dominant effect in the identification of enhancers.

### Performance of ChIP-Seq-based feature groups and the combination of chromatin state and sequence-based feature groups

Generally, enhancers are characterized according to the binding sites of different histones, such as H3K4me1, H3K27ac and H3K27me3 11. The results in Table 2 indicate that the model incorporating chromatin state demonstrated good performance consistent with that in previous publications^11^,^13^,^16^.

In addition to the models described above, we also constructed a model based on the Reads Per Million mapped reads per base pair densities (RPM) of 61 TFs. The results are listed at the end of Table 2. The performance of the TF RPM-based model was superior among all models in Table 2, with an ACC of 0.9937, MCC of 0.9874 and AUC of 0.9989. It has been suggested that TF binding signals play a crucial role in the identification of enhancers. When we considered both TF RPM, which was based on experimental data, and TF binding motif occurrence, which was based on sequence data, both models suggested that TFs play a very important role in the identification of enhancers. Because the model incorporating TF RPM features required 61 transcription factor ChIP-Seq datasets and achieved near-perfect performance, it was not combined with other features in the following analyses.

For the combinations of chromatin state features and sequence-based features, the results indicate that the models incorporating the TF binding motif occurrence or chromatin state feature group generally demonstrated good performance. The model with the best performance incorporated the combinatorial features of TF binding motif occurrence and chromatin state and achieved an ACC of 0.9466, MCC of 0.8937and AUC of 0.9882, which was better than any single feature group involving TF binding motif occurrence and chromatin state. This result indicates a complementary effect between chromatin state and TF binding motif occurrence, implying that both chromatin state and TF binding should be considered in the studies of enhancer functions. The results also indicate that different combinations of different feature groups result in differential performance, and the combinational feature groups usually demonstrate better performance than single feature groups. However, the model incorporating the largest number of features (DNA properties, TF binding motif occurrence, k-mers and chromatin state) did not exhibit the best performance. These results suggest that good-quality features with proper characterization are the most important component for the identification of enhancers, and increasing the number of features does not always result in improved performance, because new features may introduce noise into the model and consequently worsen the performance.

### Feature selection and the performance of different selected feature sets

**Figure 1:**
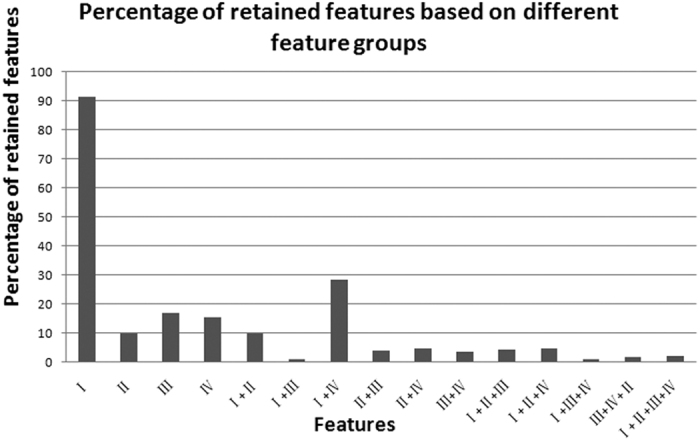
Percentage of selected features across different feature groups. The *x*-axis represents different feature groups and their combinations. The *y*-axis represents the percentage of selected features. I represents DNA property features; II represents TF binding motif occurrence features; III represents k-mer features; IV represents chromatin state features.

Although the models in Tables 1 and 2 generally demonstrated good performance, they are built on many features. Per the Pareto criterion, a good model possesses a larger fitness metric, such as ACC, MCC or AUC, and a parsimony metric, such as a smaller number of features^27^. To reduce the number of features and improve the performance, the varSelRF package was used to select the informative features^28^ from all models in Tables 1 and 2. The updated results for all of the models based on the selected features are given in Table 3. When the performance of models in Table 3 was compared with the performance of the corresponding models in Tables 1 and 2, fitness metrics such as ACC, MCC or AUC were relatively stable or improved, although the number of features was notably smaller. When the number of features in Table 3 was compared with the features of the corresponding models in Tables 1 and 2, only a small fraction of features were important for the identification of enhancers. The percentage of selected features in different feature groups is shown in Fig. 1. In particular, only 141 (~10%) features in the TF binding motif occurrence feature group were retained without performance deterioration. This result indicates that the majority of features, even 80% or 90%, can be removed, suggesting that most features have little effect on the identification of enhancers.

**Figure 2:**
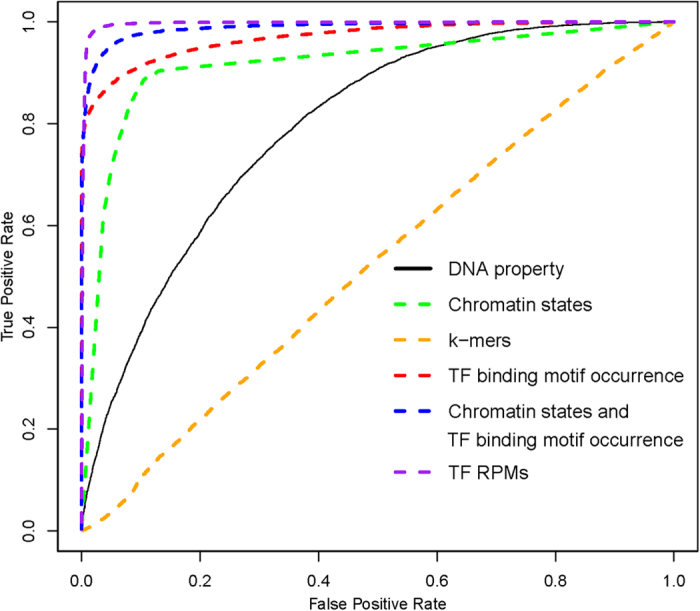
The receiver operator characteristic (ROC) curves for selected feature groups. Area under the ROC curve (AUC): DNA property features (black line): 0.793; TF binding motif occurrence features (red dashed line): 0.9698; k-mer features (orange dashed line): 0.5213; chromatin state features (green dashed line): 0.9159; chromatin state and TF binding motif occurrence features (blue dashed line): 0.989; TF RPM features (purple dashed line): 0.9964. TF RPM represents the Reads Per Million mapped reads per base pair densities (RPM) of ChIP-Seq data from 61 TFs.

When we compared the performance of all models in Table 3, models based on the feature groups for chromatin state, TF binding motif occurrence, the combination of both chromatin state and TF binding motif occurrence, and TF RPM usually demonstrated better performance. Among these 4 models, the model based on the TF binding motif occurrence feature group used only sequence information. The other three models used experimental information, including corresponding ChIP-Seq datasets when available. The ROC curves of selected feature groups are given in Fig. 2. The results clearly showed that the model with transcription factor binding motif occurrence features achieved a performance with an AUC of 0.9698, which was comparable to the performance of models with ChIP-Seq-based features. This result indicates that it is reasonable to predict enhancers via sequence-based features alone. The number of selected features in the model based on the feature group of TF binding motif occurrence was 141. There are nearly 1,900 known TFs in humans^29^, and most of them are conserved in mice^29^. However, only a small fraction of these TFs are important in the identification of enhancers, thus suggesting that TFs are involved in very complex mechanisms in the context of enhancer function.

The model incorporating the feature groups for TF binding motif occurrence and chromatin state demonstrated the best performance in Table 3, achieving an AUC of 0.989 and ACC of 0.9525, with the exception of the TF RPM model. This performance was better than that of any model for a single feature group such as TF binding motif occurrence or chromatin state. Although the number of features in the model based on chromatin state was only 10, its performance achieved an ACC of 0.8905, MCC of 0.781 and AUC of 0.9159. These results indicate that chromatin state is important for the identification of enhancers. In addition, the number of features in the model based on the combinational feature group of TF binding motif occurrence and chromatin state was much smaller than the number of features in the model based on the feature group of TF binding motif occurrence. These findings clearly demonstrate a complementary effect between chromatin state and TFs, which is consistent with a recent publication stating that enhancers not only are a collection of TF binding sites but also are enriched in certain chromatin states^14^. All of the above results indicate that it is possible to predict enhancers on the basis of a combination of TF binding motif occurrence and chromatin state.

In addition, we applied the varSelRF method to select informative features according to TF RPM signals. The performance of the model with 28 selected TF RPM features achieved an ACC of 0.994, MCC of 0.998 and AUC of 0.9985. Because several TFs were also used to define enhancers such as Oct4, Sox2, Nanog and Med1 in previous publications^8^,^30^, we excluded these 4 TFs, and the rebuilt model still demonstrated a performance achieving an ACC of 0.9802, MCC of 0.9605 and AUC of 0.9964. The specific results are listed at the end of Table 3. Among the remaining 24 TFs, it should be noted that p300 was included; however, the binding sites of p300 are generally considered to be enhancers^19^,^31^. Thus, p300 was also excluded, and the model was rebuilt with the remaining 23 TFs. The performance of this model achieved an ACC of 0.9871, MCC of 0.9613 and AUC of 0.9966. These results implied that enhancers are enriched in many TFs, not only those used in previous studies^8^,^30^ but also many others that are not fully understood. Furthermore, there is a great deal of crosstalk between different TFs.

Again, it is clear that only a small fraction of TFs are important in the identification of enhancers. Moreover, these selected TFs may possess important roles in enhancer function.

### Importance of selected features

**Figure 3:**
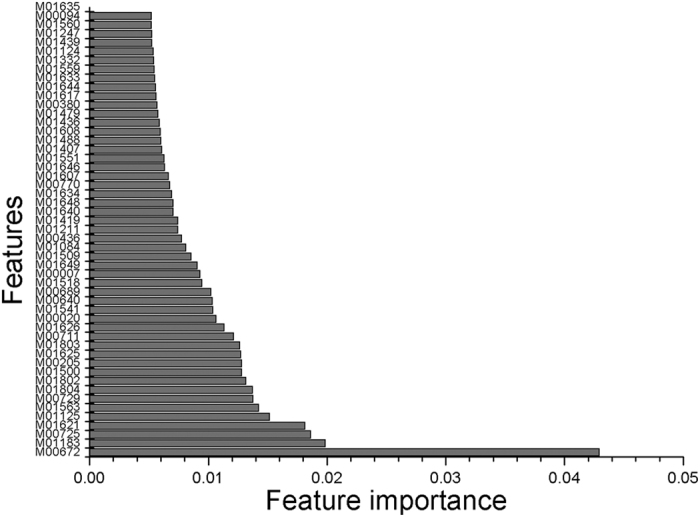
Importance of the model incorporating TF binding motif occurrence features. The importance of the top 50 selected features in the model with 141 TF binding motif occurrence features is shown. The prefix M represents Position Weight Matrix (PWM).

**Figure 4:**
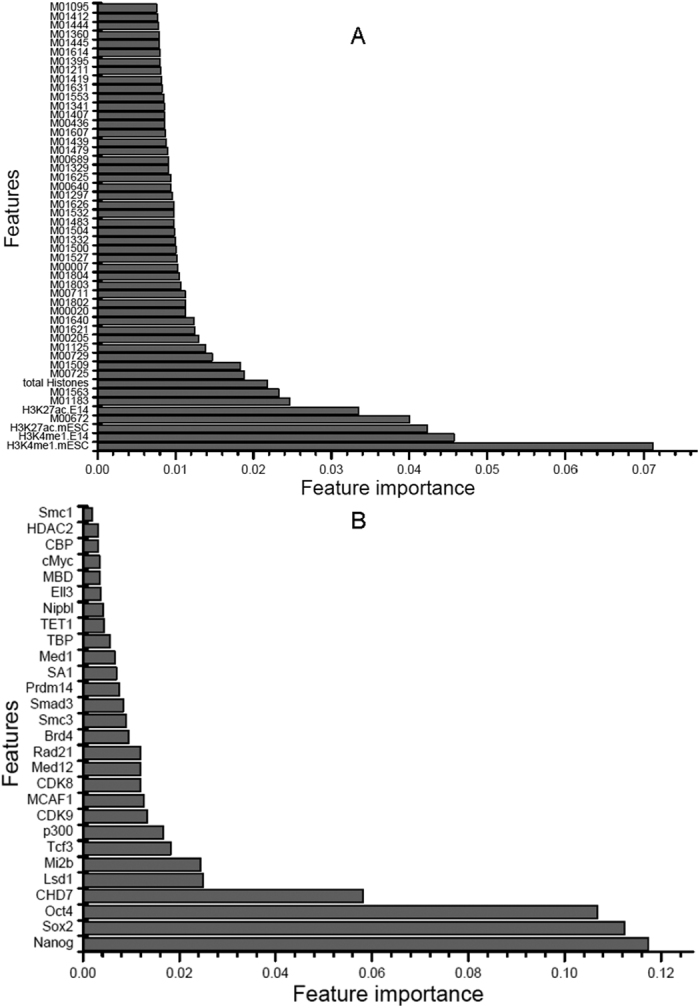
Importance of selected features. (**A**) shows the importance of the top 50 selected features in the model with the feature groups for TF binding motif occurrence and chromatin state in Table 3. (**B**) shows the importance of the 28 selected features in the model based on the feature group TF RPM in Table 3. TF RPM represents the Reads Per Million mapped reads per base pair densities (RPM) of ChIP-Seq data from 61 TFs. The prefix M represents Position Weight Matrix(PWM).

**Figure 5:**
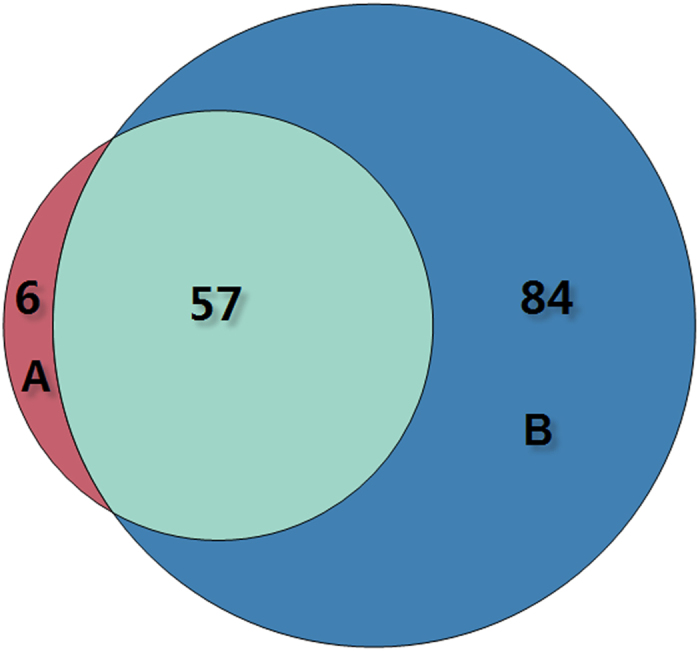
Venn diagram of the selected TF binding motif occurrence features of the model incorporating the feature group TF binding motif occurrence and the model incorporating the feature groups of TF binding motif occurrence and chromatin state. A represents selected TF binding motif occurrence features of the model incorporating the feature group TF binding motif occurrence and chromatin state. B represents selected TF binding motif occurrence features of the model incorporating the feature group TF binding motif occurrence.

To further evaluate the importance of our selected features, a permutation method implemented in the R package rfPermute^32^ was applied. The importance of the top 50 features in the TF binding motif occurrence model is shown in Fig. 3. The importance of all 141 selected features is provided in Supplementary Figure S1A. In addition, according to the feature importance, the top 50 features of the model incorporating the feature groups for TF binding motif occurrence and chromatin state are also shown in Fig. 4A, and the full list of all selected features can be found in Supplementary Figure S1B. A Venn diagram is given in Fig. 5 comparing the model incorporating TF binding motif occurrence and the model incorporating both TF binding motif occurrence and chromatin state. Among the 63 TF binding motif occurrence features in the model based on the feature group incorporating TF binding motif occurrence and chromatin state, 57 features overlapped with the 141 selected features from the model based on TF binding motif occurrence. This finding demonstrates that most features from the model incorporating TF binding motif occurrence and chromatin state overlapped with the model incorporating TF binding motif occurrence.

Figure 4A indicates that most of the selected features corresponded to TF binding motif occurrence, whereas only 5 features comprised histone modifications. These histone modifications are consistent with the results of a previous publication showing that active enhancers are usually enriched in H3K4me1 and H3K27ac binding sites^33^. Although most individual TF binding sites have little effect on the identification of enhancers, their combination exerts much more important effects than any sites from single TFs. Comparison of the importance of different feature groups in Fig. 4A indicated that a single chromatin state feature often plays a more important role than a single TF binding motif occurrence feature for enhancer identification. However, this is not the case for combinations of chromatin state and TF binding motif occurrence: their integration can greatly improve enhancer identification.

Based on the experimental TF RPM datasets, the TFs selected by the varSelRF method were Brd4, CBP, CDK8, CDK9, CHD7, cMyc, Ell3, HDAC2, Lsd1, MBD, MCAF1, Med1, Med12, Mi2b, Nanog, Nipbl, Oct4, p300, Prdm14, Rad21, SA1, Smad3, Smc1, Smc3, Sox2, TBP, Tcf3 and TET1. Their relative importance is indicated in Fig. 4B. It has been suggested that these TFs are important for enhancer functions. For example, the TFs Nanog, Oct4 and Sox2 demonstrate the greatest feature importance in Fig. 4B and have been used to define enhancer regions in previous publications^8^,^30^. Furthermore, they are master transcription factors and play important roles in cell differentiation. After excluding all TFs used to define enhancers, the rebuilt model still demonstrated good performance (see Table 3). In addition, many other general enhancer-related TFs were identified, such as Brd4, CBP, CHD7, cMyc, HDAC2, Lsd1, MBD, Med12, Mi2b, Nipbl, p300, Prdm14, Smad3, Smc1 and Smc3, which are cofactors involved in enhancer functions^8^. p300 binding sites are generally considered to be enhancers^19^,^31^. After exclusion of p300 binding sites, the performance of the rebuilt model was still retained. A recent publication^34^ has indicated that CDK8 and CDK9 are involved in the functions of super-enhancers. The Ell3 protein is an elongation factor that can bind to enhancers^35^. The TET1 protein is related to DNA methylation^36^ and controls enhancer functions^15^. Recent studies have also revealed that other TFs, such as MCAF1^37^, SA1^38^, Rad21^38^,^39^, TBP^39^ and Tcf3^40^, play roles in enhancer functions. In particular, our recently published results have revealed that cohesin complex components Rad21 and Smc3 exhibit co-occupancy with CCCTC-binding factor (CTCF)^41^, which plays important roles in the structures of enhancer-promoter loops. These results indicate that enhancers are associated with a variety of TFs and suggest that there are synergistic effects among different TFs; thus, special attention should be paid to the co-regulation of these selected TFs.

### Comparison with previous methods and application to other datasets

There are several methods for predicting enhancer activities, such as the logistic regression model^13^, Bayesian network^22^, random forest^16^ and SVM^11^. Both Rajagopal *et al*.^16^ and Zhu *et al*.^13^ have used histone modification features to predict enhancers. The results from these methods are shown in Table 4; certain criteria were not available for certain methods. Among the three criteria in Table 4, MCC is the most rigorous. Table 4 shows that our methods were superior to previous methods for all three criteria.

Previous studies have indicated that k-mers, particularly gapped k-mers, can be effectively used to predict enhancers^21^. To further compare k-mer approaches, our method was applied to mouse embryo brain and limb datasets comprising p300 protein binding sites. For the mouse embryo brain dataset, our method achieved a performance of AUC 0.8742, which was somewhat less rigorous than the previously reported performance of 0.94^42^. For the mouse limb dataset, our method achieved a performance of AUC 0.8738, which was also somewhat less rigorous than the previously reported performance of 0.91^42^. The value of k in the k-mers used in a previous study^42^ ranged from 3 to 10, a larger range than that achieved with our k-mer method. An improved k-mer method, the gapped k-mer approach^21^, has been applied to identify p300 protein binding sites and has demonstrated a performance of AUC 0.947^21^. When our method was applied to this dataset, it achieved a performance of AUC 0.8643. Both ROC and precision-recall curves are given in Supplementary Figure S2.

In addition, the gapped k-mer method was also applied to our datasets, and the results are listed in Table 4. It achieved a performance of ACC 0.8531 and AUC 0.9311, which is somewhat poorer than our method. All of those results indicate that our TF binding motif occurrence method demonstrates good performance for enhancer prediction and may be superior or comparable to the gapped k-mer method.

Different datasets were used to further test the performance of the sequence-based model. The first dataset encompassed enhancer and heterochromatin regions generated by ChromHMM^43^ according to chromatin state and transcription factors. The ChromHMM^43^ method is a clustering-based method to annotate the regulatory regions of the genome, and it was applied to murine embryonic stem cells (mESCs) based on the available chromatin state ChIP-Seq datasets for H3K4me1, H3K4me2, H3K4me3, H3K9ac, H3K27ac, H3K27me3 and H3K36me3 as well as of the RNA polymerase II (Pol II) and CTCF proteins. In total, 43,675 enhancers and 13,282 heterochromatin regions were annotated on the basis of histone modification and transcription factor ChIP-Seq signals. Among the 43,675 enhancers, 6,855 (15.7% accuracy) enhancers from ChromHMM could be mapped to the 10,627 enhancers used in the current work. Additionally, 5,990 of 10,627 enhancers (56.37% coverage) uniquely mapped to the 43,675 ChromHMM predicted enhancers.

For testing purposes, the model comprising 141 TF binding motif occurrence features was applied to predict all 43,675 enhancers. The results showed that 43,564 (99.75%) enhancers were correctly identified. Furthermore, the model was applied to the heterochromatin regions predicted by ChromHMM. Among the 13,282 heterochromatin regions, 10,869 (81.83%) were identified as non-enhancer regions. Thus, the model comprising only the 141 selected TF binding motif occurrence features could be used to identify both enhancer and non-enhancer regions.

The second dataset consisted of mouse enhancers from the Vista enhancer project. We downloaded all 568 mouse enhancers from the Vista enhancer project (http://enhancer.lbl.gov/) website. Among these mouse enhancers, only 18 enhancers could be mapped to the 10,627 enhancers in the current work. When the model with the 141 selected TF binding motif occurrence features was applied to this dataset, 566 (99.65%) were correctly identified as enhancers. Given that many enhancers in the Vista enhancer project are tissue-specific, this result indicates that the model with the 141 TF binding motif occurrence features could be applied to identify enhancers in other cell lines/tissues.

In addition, we tested our model on human enhancer datasets. There are 68 enhancer datasets from different human cell lines/tissues available from the FANTOM5 project^11^. We downloaded all 68 enhancer datasets and applied our model with 141 TF binding motif occurrence features without changes. The prediction accuracy ranged from 93.15% to 100%, and the specific results for each cell line are listed in Supplementary Table S1. These results suggest that the model with the 141 selected TF binding motif occurrence features can be used to predict enhancers not only from murine embryonic stem cells (mESCs) and other cells but also from different human cell lines/tissues.

## Discussion

Enhancers play important roles in gene regulation and expression, cell differentiation, chromatin looping and 3-dimensional (3D) genome structure. Enhancers generally carry out their functions together with promoters and recruit various TFs and histone modifications to assist in these processes. In this work, we systematically investigated various sequence-based feature sets, such as DNA properties, k-mers, TF binding motif occurrence and their combinations. The results indicated that the performance of the model incorporating TF binding motif occurrence was best, followed by DNA properties and k-mer features. Then, a recursive feature elimination method was applied to select the most informative features. In most cases, 80% or even 90% of features could be removed without significantly affecting fitness metrics, such as ACC, MCC and AUC.

We tested our sequence-based model incorporating 141 selected TF binding motif occurrence features on different datasets. The results based on datasets derived from the mouse ChromHMM chromatin state, FANTOM enhancers, and human enhancers in different cell lines showed that our model was applicable to different cell lines and two different species. Thus, our model is robust for generalized enhancer prediction.

When ChIP-Seq-based features were included, the best model incorporated both TF binding motif occurrence and the chromatin state and achieved an ACC of 0.9525, AUC of 0.989 and MCC of 0.905. This model contained only 69 features, of which 6 were chromatin state features and 63 were TF binding motif occurrence features. The results indicate that, of the 1422 available TF binding PWMs, only a small fraction of TF binding sites are important in enhancer identification. Selected features, such as TFs and chromatin state, are known to play various important roles in determining the functions of enhancers. Our results also show that enhancers can be identified by integrating both TF binding motif occurrences and chromatin state. There are complementary effects between TFs and chromatin state. A single TF exerts a minor effect on the identification of enhancers. However, the combination of TFs can have a determinant effect on enhancer functions, thus implying that special attention should be paid to TFs in addition to chromatin state in studies of enhancer functions. Our methods represent alternative ways to study the functions of enhancers and may be complementary to wet-lab experimental methods.

## Methods

### Datasets

A total of 10,627enhancers, considered positive samples, from murine embryonic stem cell (mESCs) were collected from previous publication^8^. Enhancer regions were defined as the regions enriched in H3K27ac and TFs, such as Oct4, Sox2, Nanog and Med1^8^,^30^. Random genomic regions of equivalent sizes to the enhancers, used as negative samples, were generated via random shifts on the same chromosome^8^ and were also collected from previous publication^8^. In total, there were 21,254 enhancers and control regions in the final dataset. The sequence of each region was extracted from the *mm9* mouse reference genome. These datasets are given in Supplementary Dataset S1 in bed format. To improve the predictive performance of enhancers, transcription factor ChIP-Seq datasets were also collected from previous publication^8^.

### Feature construction

In this work, features were divided into two categories: sequence-based and ChIP-Seq-based. The sequence-based features included the following three groups: DNA properties, k-mers and transcription factor binding motif occurrences. In total, 4,343 individual features, which are summarized in Table 5, are described in the following subsections, Group I to Group V. In addition to sequence-based features, we used available ChIP-Seq datasets to generate ChIP-Seq-based features. These features were considered for situations in which histone modification or transcription factor ChIP-Seq datasets were available. Detailed information for each of these feature groups is described in the subsections under Group IV and Group V.

### DNA property-based features (Group I)

For a given enhancer or control region, the DNA properties were calculated by using the structural properties of di- or tri-nucleotides with a corresponding sliding window width of 2 or 3 along the DNA sequence throughout the region. Then, the average of each property was calculated as the final DNA property. In total, we collected 23 structural properties of nucleotides, which were used in previous publications^31^,^44^. These 23 properties were calculated on the basis of experimental data or molecular modeling of a DNA helix or a DNA-protein complex. Thus, this feature group, which characterizes the structure of a DNA molecule, was designated “DNA properties” and contained 23 features.

### TF binding motif occurrence-based features (Group II)

Previous studies have shown that DNA regulatory regions are occupied by many TFs^2^,^22^. Generally, the binding sites of TFs can be characterized by Position Weight Matrix (PWM). In this work, a total of 1,422 PWMs were collected from Cistrome^45^ and the TRANSFAC track in the UCSC Genome Browser^46^. For a given sequence, the ability of a TF to bind DNA was represented by the transcription factor affinity prediction (TRAP) score^47^, which was calculated according to the transcription factor motif PWM by the TRAP^47^ program. The parameters of the TRAP program used default values. Because one PWM generates a TRAP score, there were 1,422 TRAP scores for a given sequence. This feature group was designated “TF binding motif occurrence” and contained 1,422 features.

### k-mer-based features (Group III)

K-mer features of sequences are widely used for coding DNA^44^,^48^ and RNA^49^. The value of k is important in this method. In a previous study, k was systematically evaluated from 1 to 6, and the relative importance of each k-mer feature was assessed; ultimately, 2,772 k-mers were retained^44^. In this work, the composition of these 2,772 k-mers was calculated for each sequence of an enhancer or control region. Thus, this feature group was designated “k-mers” and contained 2,772 features.

### Chromatin state-based features (Group IV)

In this work, histone modification datasets from mice were collected from the ENCODE Project^12^, which catalogs 64 histones from different mouse tissues. For a given enhancer or control region, 1 was indicated if the region contained a histone modification peak; otherwise, the region was encoded as 0. This procedure was repeated for the other 63 histone modification datasets. For a given region, the summary feature was the total number of histone modifications across all 64 histone modification datasets. Thus, this feature group was designated “chromatin state” and contained 65 features.

### TF RPM-based features (Group V)

Transcription factor ChIP-Seq datasets were collected from previous publications^8^. Enhancer and control regions were characterized by the Reads Per Million mapped reads per base pair densities (RPM) from ChIP-Seq data from 61 TFs. For each enhancer and control region, the final densities were calculated via the subtraction of the RPM of a given TF from the RPM of the corresponding input file for that TF. Thus, this feature group was designated “TF RPM” and contained 61 features.

### Machine learning method and feature selection

**Figure 6:**
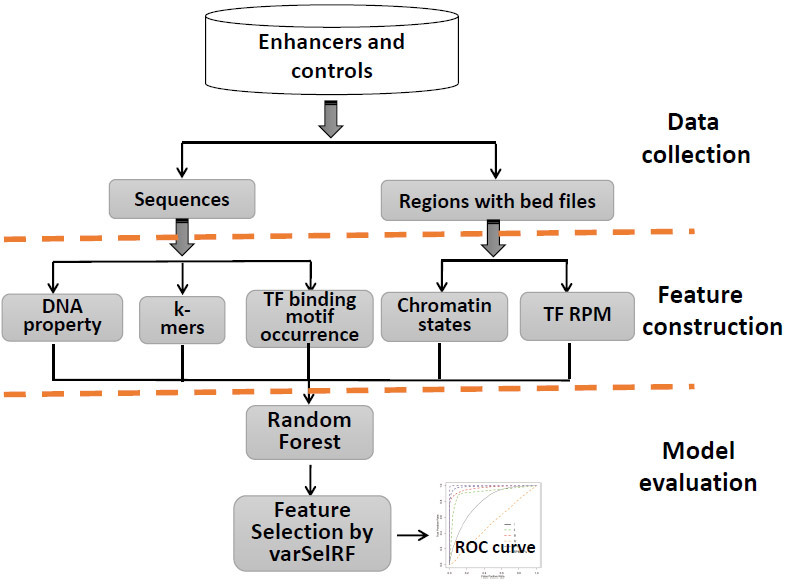
Overall schematic of this work.

Random forest is an ensemble method based on decision trees in which each tree is constructed independently of a data subset^50^. Our previous work^51^,^52^ has indicated that random forest and Support Vector Machine (SVM) usually demonstrate good performance with various datasets. This finding is consistent with the recently published work of Fernandez-Delgado *et al*.^53^, who have systematically evaluated 179 classifiers across 121 UCI datasets, and have found random forest to be the best family of classifiers. In this work, random forest was applied because it is generally more robust than SVM, which is a parameter-sensitive method and requires a long period of time to optimize parameters. The random forest package in R software was used in this study, as in our previous study^52^. The *ntree* parameter was set to 5,000, which historically has demonstrated good performance^51^,^52^, and the importance was set to TRUE. To build a robust model, the Pareto optimization rule^27^ was applied, which favors a good model with better performance and fewer numbers of features. The varSelRF R package was used to select informative features^28^; this package includes a recursive feature elimination method and utilizes feature importance for feature evaluation and selection. The drop fraction in each iteration was set to 0.1. Other parameters were set to default. To further evaluate the relative importance of the selected features, another R package, rfPermute^32^, was used, which is a feature importance evaluation method that permutes the response variable. The number of permutation replicates was set to 100, and *ntree* was set to 5,000. The average decreasing accuracy was used to evaluate the feature importance. An overall schematic of our work is shown in Fig. 6.

### Performance evaluation

The performance of all models was evaluated with 10-fold cross-validation. Specifically, the enhancers and control regions were divided into 10 groups of nearly equal size. One group of enhancers and one group of control regions were then taken together as the testing dataset, and the others were used as the training dataset. This procedure was repeated until each group of enhancers and control regions was taken as the testing set once. To assess the performance of the built models, several metrics were used and are given below.


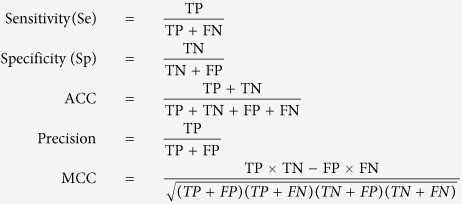


where TP, TN, FP and FN are true positive, true negative, false positive and false negative, respectively. ACC indicates accuracy. Sensitivity is referred to as the true positive rate and is also referred to as recall. The Mathews correlation coefficient (MCC) is a measure of the correlation coefficient between the observed and predicted binary classifications. The parameter MCC is more rigorous than ACC. The receiver operating characteristic (ROC) curve is a graphic plot of sensitivity against the false-positive rate (1-specificity). The area under an ROC curve (AUC) demonstrates the trade-off between sensitivity and specificity. The value of AUC is within the range of 0 to 1. An AUC of 0.5 represents random classification, and an AUC of 1 indicates perfect prediction.

## Additional Information

**How to cite this article**: Fang, Y. *et al*. In silico identification of enhancers on the basis of a combination of transcription factor binding motif occurrences. *Sci. Rep.* **6**, 32476; doi: 10.1038/srep32476 (2016).

## Supplementary Material

Supplementary Information

Supplementary Information

## Figures and Tables

**Table 1:** Performance of models built on sequence-based feature groups.

Feature groups	# of features	Se	Sp	ACC	MCC	AUC
DNA property(I)	23	0.7759	0.6675	0.7217	0.4460	0.7943
**TF binding motif occurrence(II)**	**1422**	**0.8203**	**0.9783**	**0.8993**	**0.8087**	**0.9687**
k-mers(III)	2772	0.5197	0.4695	0.4946	−0.0108	0.5024
I + II	1445	0.8256	0.9785	0.9020	0.8136	0.9703
I + III	2795	0.8050	0.5962	0.7006	0.4103	0.7806
II + III	4194	0.8170	0.9739	0.8955	0.8008	0.9678
I + II + III	4217	0.8190	0.9753	0.8972	0.8043	0.9699

I represents DNA property features; II represents TF binding motif occurrence features and III represents k-mer features.

**Table 2:** Performance of models with ChIP-Seq-based features added.

Feature groups	# of features	Se	Sp	ACC	MCC	AUC
**Chromatin state(IV)**	**65**	**0.8883**	**0.8939**	**0.8911**	**0.7822**	**0.9174**
**II + IV**	**1487**	**0.9324**	**0.9609**	**0.9466**	**0.8937**	**0.9882**
II + III + IV	4259	0.9184	0.9657	0.9421	0.8852	0.9874
I + II + IV	1510	0.9332	0.9612	0.9472	0.8948	0.9884
I + II + III + IV	4282	0.9183	0.9650	0.9417	0.8843	0.9871
TF RPM	61	0.9965	0.9909	0.9937	0.9874	0.9989

I represents DNA property features; II represents TF binding motif occurrence features; III represents k-mer features; IV represents chromatin state features; TF RPMs represent the Reads Per Million mapped reads per base pair densities (RPM) of ChIP-Seq data from 61 TFs.

**Table 3:** Performance of models built on different selected feature sets.

Feature groups	# of features	Se	Sp	ACC	MCC	AUC
DNA property(I)	21	0.7734	0.6683	0.7209	0.4442	0.793
**TF binding motif occurrence(II)**	**141**	**0.8473**	**0.9753**	**0.9113**	**0.8293**	**0.9698**
k-mer(III)	463	0.5559	0.4912	0.5235	0.4724	0.5213
Chromatin state(IV)	10	0.8878	0.8933	0.8905	0.7810	0.9159
I + II	141	0.8545	0.9724	0.9135	0.8328	0.9711
I + III	22	0.7760	0.6669	0.7215	0.4456	0.795
II + III	160	0.8468	0.9776	0.9122	0.8316	0.9697
**II + IV**	**69**	**0.9550**	**0.9500**	**0.9525**	**0.9050**	**0.989**
I + II + III	179	0.8533	0.9749	0.9141	0.8344	0.9711
II + III + IV	77	0.9537	0.9514	0.9525	0.9050	0.9894
I + II + IV	71	0.9519	0.9502	0.9511	0.9021	0.9891
I + II + III + IV	87	0.9183	0.9650	0.9417	0.8843	0.9891
**TF RPM**	**24**	**0.9869**	**0.9735**	**0.9802**	**0.9605**	**0.9964**

I represents DNA property features; II represents TF binding motif occurrence features; III represents k-mer features; IV represents chromatin state features; TF RPM represent the Reads Per Million mapped reads per base pair densities (RPM) of ChIP-Seq data from 61 TFs.

**Table 4:** Comparison of our model with previously reported models.

Method (Reference)	ACC	AUC	MCC
Zhu *et al*.^13^	—	0.935	0.712
RFECS^16^	0.828	—	—
Taher^11^	—	0.93	—
BNFinder^22^	—	0.93	—
Gapped k-mers^19^	0.8531	0.9311	0.7065
**Our method**	**0.9113**	**0.9698**	**0.8293**
**Our method**^**a**^	**0.9525**	**0.989**	**0.905**

Superscripts represent the model based on the combination of TF binding motif occurrence and chromatin state features in Table 3.

**Table 5:** The list of 4,343 features.

	Group	Features	Number of features	Source
Sequence-based features	I	DNA property	23	In-house script
	II	TF binding motif occurrence	1,422	45, 46
	III	k-mers	2,772	44, 48
ChIP-Seq-based features	IV	Chromatin state	65	12
	V	TF RPM	61	8
