# Supplementary material for: In silico identification of enhancers on the basis of a combination of transcription factor binding motif occurrences
Source: Sci Rep. 2016 Sep 1;6:32476. doi: 10.1038/srep32476 (PMC5007594; doi:10.1038/srep32476)
Supplement: Supplementary Information [file srep32476-s1.pdf]

Supplementary Information

In silico identification of enhancers on the basis of a combination of transcription factor binding motif occurrences

Yaping Fang[1,2]*, Yunlong Wang[1,2], Qin Zhu[1,2], Jia Wang[1,2], Guoliang Li[1,2,3]*

[1]Agricultural Bioinformatics Key Laboratory of Hubei Province, [2]College of Informatics,

[3]National Key Laboratory of Crop Genetic Improvement, Huazhong Agricultural University, Wuhan 430070, China.

*To whom correspondence should be addressed.

Email: ypfang@mail.hzau.edu.cn, guoliang.li@mail.hzau.edu.cn

Supplementary Figures
Supplementary Figures S1
Supplementary Figures S2

Supplementary Tables
Supplementary Table S1

Supplementary Dataset
Supplementary Dataset S1

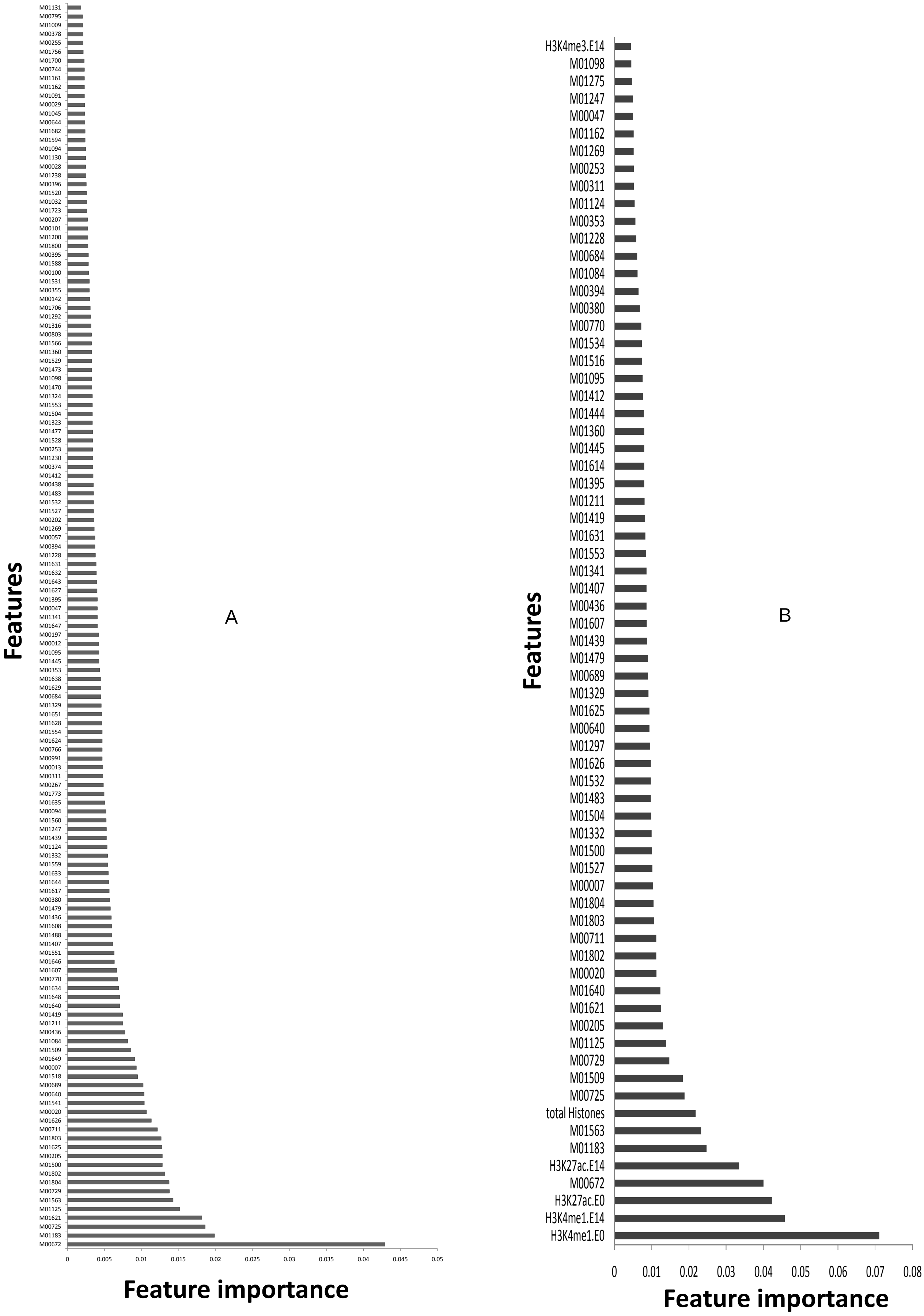

Figure S1. Importance of selected features. Figure S1A represents the importance of the selected features in the model incorporating TF binding motif occurrence features in Table 3. Figure S1B represents the importance of the selected features in the model incorporating the feature group TF binding motif occurrence and the chromatin state in Table 3. The prefix M represents Position Weight Matrix (PWM).

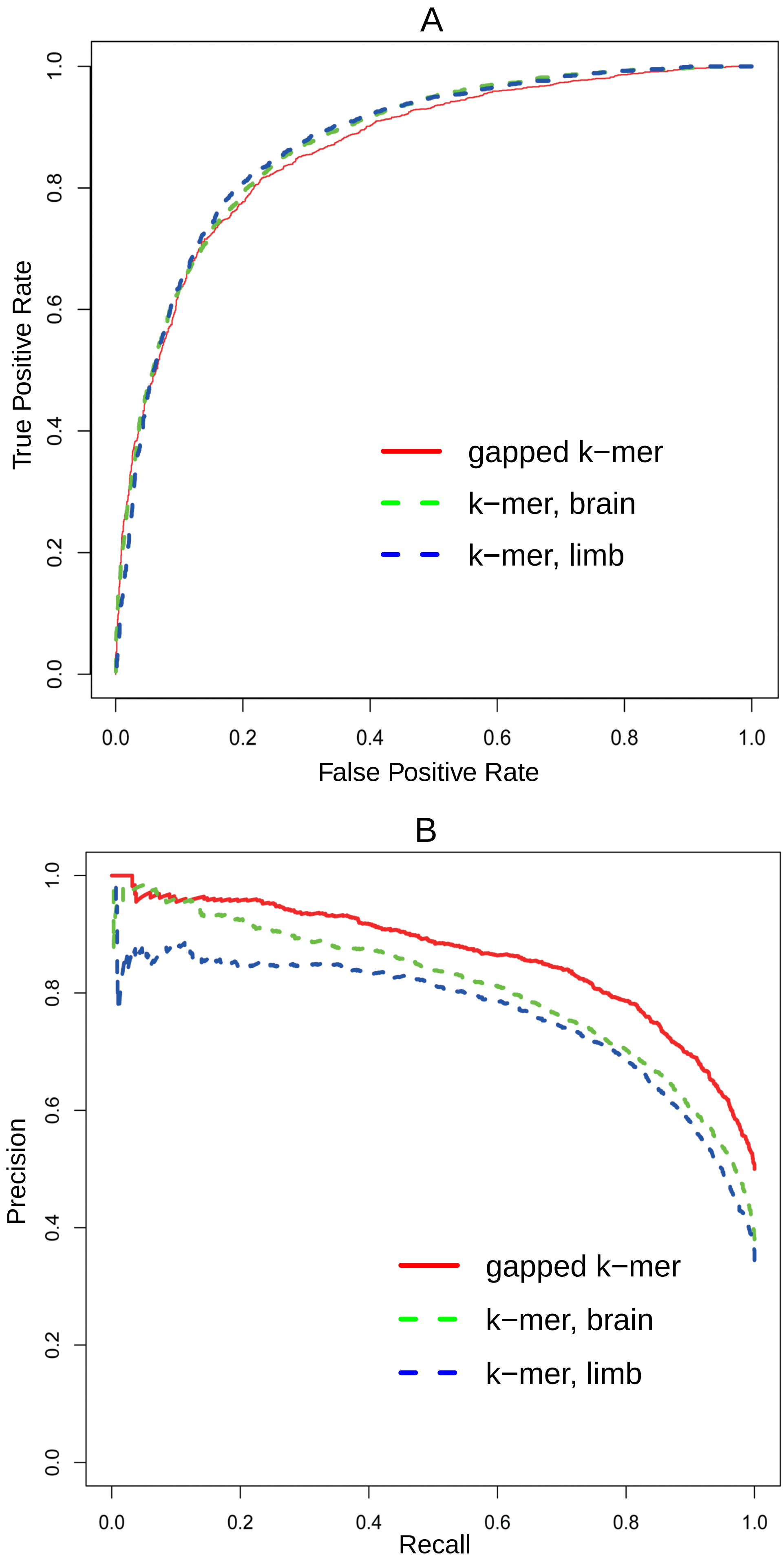

Figure S2. The receiver operator characteristic (ROC) and precision-recall curves for different datasets. Figure S2A represents the ROC curves for different datasets. Figure S2B shows the precision-recall curves for different datasets.The value of AUC is 0.8742 for the mouse embryo brain dataset (green dash line), 0.8738 for the mouse limb dataset (blue dash line) and 0.8643 for the binding sites of P300 protein (red line).
